# Transapical Closure of Re-entry Tear in Aortic Dissection With the Off-Label Use of a Gore Cardioform Septal Occluder Using 3D-Printed Simulation

**DOI:** 10.1016/j.cjco.2025.04.010

**Published:** 2025-04-20

**Authors:** Christoph Huber, Louis Vaisière, Hajo Müller, Christoph Ellenberger, Mustafa Cikirikcioglu, Jean Paul Vallée, Stephane Noble

**Affiliations:** a Cardiovascular Surgery Division, Department of Surgery, Geneva University Hospital and University of Geneva Medical School, Geneva, Switzerland; b Division of Cardiology, Department of Medicine, Geneva University Hospital and University of Geneva Medical School, Geneva, Switzerland; c Anesthesiology Division, Department of Acute Medicine, Geneva University Hospital and University of Geneva Medical School, Geneva, Switzerland; d Radiology Division, Department of Diagnostics, Geneva University Hospital and University of Geneva Medical School, Geneva, Switzerland

**We report a novel transapical approach to treat a challenging residual aortic dissection following prior type A dissection repair. Because of the complex anatomy and high surgical risk, a multidisciplinary team opted for a transapical transcatheter closure of the re-entry tear using a Gore Cardioform Septal Occluder. A custom 3D-printed model of the patient's aorta was used to simulate the device deployment. The procedure resulted in the successful exclusion of the false lumen and stabilization of the aneurysm, demonstrating the potential of this alternative technique in managing complex aortic dissections when conventional treatments are judged at too high risk.**

Residual dissection of the proximal descending aorta remains a clinical challenge after treatment of type A aortic dissection. More than half of the patients with initial type A dissection and residual dissection will present aneurysmal evolution during follow-up, with the proximal part of the distal aorta mainly affected.^1^

Currently, endovascular therapy for the residual aortic dissection in the descending aorta remains the first-line therapy.^2^ However, the location of the primary entry tear, on the aortic arch in most cases, constitute a challenge in terms of access for surgical and endovascular therapy options.^3^ This case highlights the medical application of 3D technology toward planning of a successful repair of a re-entry tear in residual aortic dissection.

## Case Presentation

**Figure 1:**
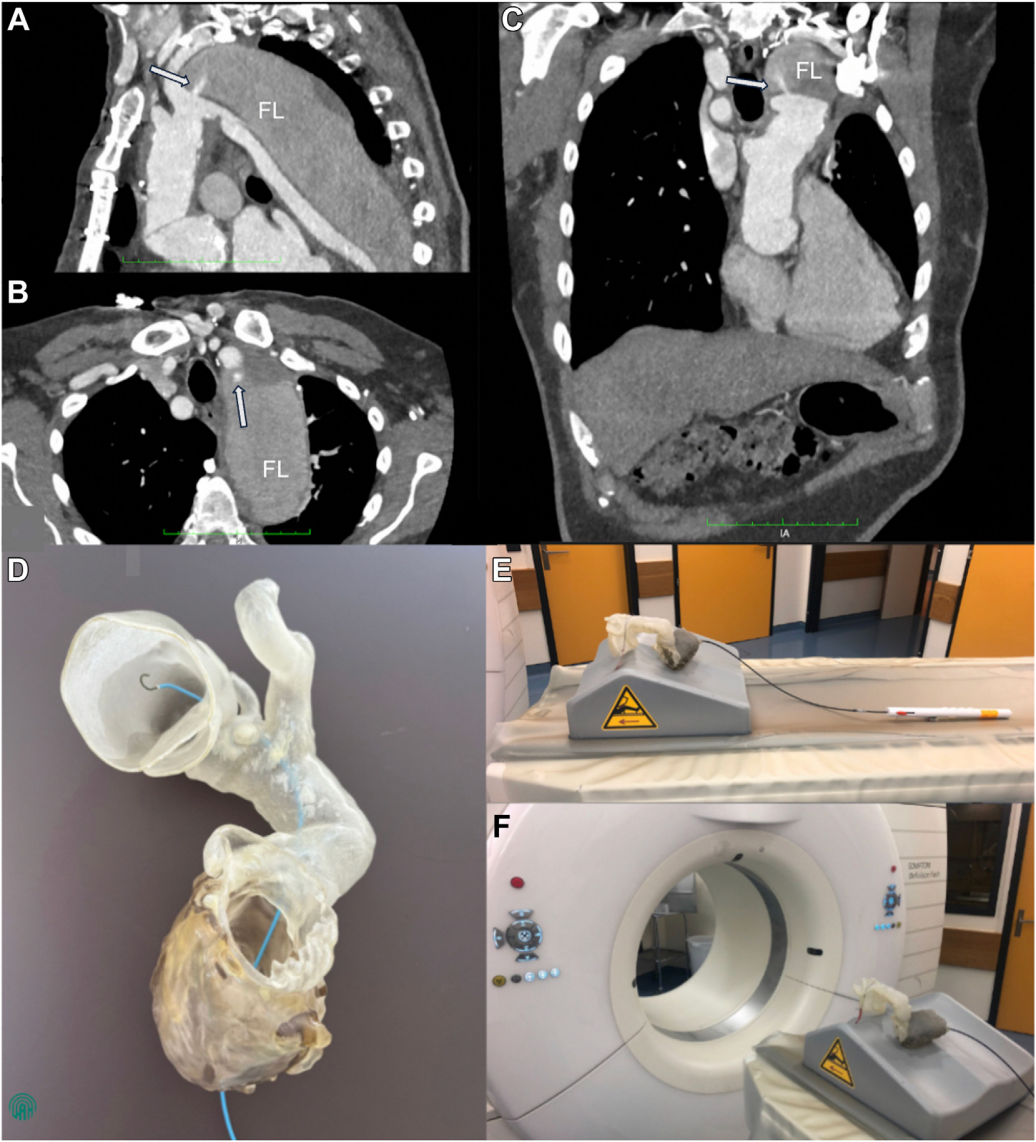
CT scan imaging of the aneurysm of the descending aorta and re-entry entry tear and the custom 3D Bench setup for preoperative planning and simulation. (**A**) Sagittal view showing the rapidly expanding aneurysm of the descending aorta (67 × 63 mm). (**B** and **C**) Coronal view and transverse view highlighting the re-entry tear in the dissection membrane at the origin of the left subclavian artery (3 mm). Note the **arrow** pointing at the re-entry tear. (**D**) Simulation of the transapical deployment and positioning of the GSO device. (**E** and **F**) In-house custom-made 3D-printed model of the patient’s heart and thoracic aorta. Ao = aorta; FL = false lumen.

A 74-year-old man with a history 3 years ago of Bio-bentall aortic root and hemiarch replacement for type A aortic dissection repair presented with a continuously expanding aneurysm within the arch and descending aorta measuring 67 × 63 mm seen with consecutive computed tomography (CT) scans at follow-up. The re-entry tear within the dissection membrane responsible for the ongoing false lumen perfusion was identified at the origin of the left subclavian artery and measured 3 mm in diameter (Fig. 1, A-C).

Considering the high surgical risk and to respect the patient's wish, a transcatheter treatment strategy was considered. After multidisciplinary discussions, we decided to attempt a transcatheter closure of the re-entry tear by inserting a closure device to obtain complete thrombosis of the proximal part of the false lumen and thus be able to stabilize the aneurysm of the proximal descending thoracic aorta. To prepare for the procedure, a custom 3D Bench setup was created for preoperative planning and simulation.

Different septal occluders have been used to treat septal defects and have shown remarkable versatility for various off-label uses, including in the context of aortic dissection.^4^^,^^5^ We opted for the off-label use of a 25-mm double-disk Gore Cardioform Septal Occluder (GSO) (WL Gore & Associates, Inc, Flagstaff, AZ) because of its biocompatible expanded polytetrafluoroethylene composition, leading to an instant sealing of the defect, allowing for periprocedural assessment of treatment success. Furthermore, the GSO device seems more flexible than the Amplatzer family (Abbott Vasc, St Paul, MN), with the latter being made with more rigid nitinol wire mesh and thus more at risk of worsening the tear as experienced in a case of false aneurysm.^6^

For proof of concept, an in-house custom-made 3D-printed model of the patient’s heart and thoracic aorta was created (Fig. 1, D and E). This model was used to simulate this novel technique before the actual intervention and to understand the deployment and position of the GSO device under radiologic control. Because of the complex residual aortic dissection, the hemiarch’s angulation, the location of the tear, and the facilitated delivery process in anterograde fashion, the decision was made to use the transapical approach for this procedure. Indeed, in addition to the unfavorable angle at which to enter the tear from the transfemoral approach, the delivery system’s working length of 75 cm was a second limitation. Considering the need for an 11-F introducer sheath, the radial or brachial approaches were not an option. The acute angulation from the left subclavian was also prohibitive. The brachiocephalic trunk was also suboptimal, considering the orientation of the GSO during deployment.

Concerning sizing, there are 3 different sizes of the GSO device (20, 25, and 30). The 20 mm was judged to be too small to fully cover the tear without risking embolization; the 30 mm was too large, with the risk of impairing the subclavian flow. We finally tested the 25-mm device on the 3D-printed model, which seemed to fit appropriately.

**Figure 2:**
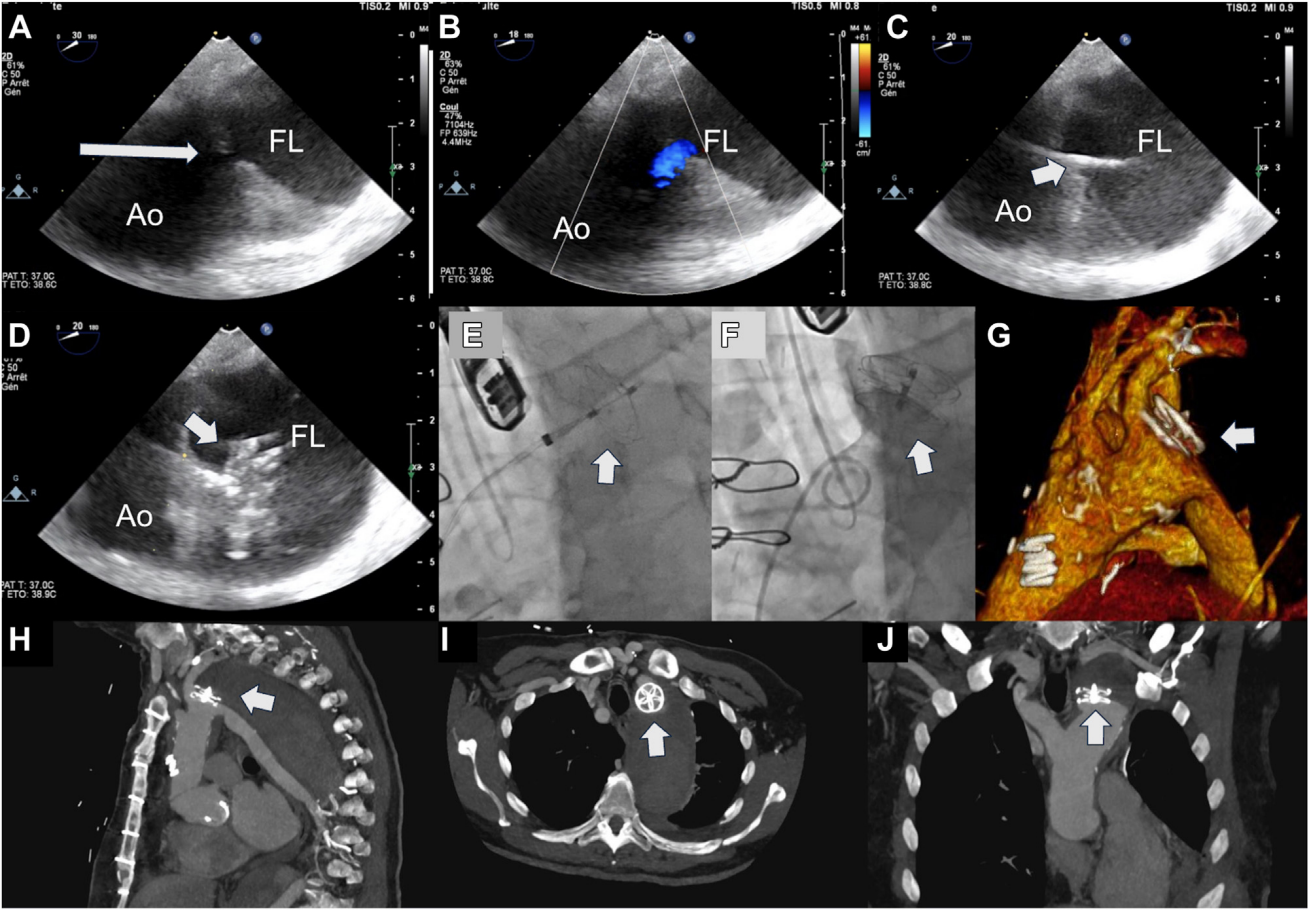
Echocardiographic and fluoroscopic images of the successful GSO device deployment and 4-day postprocedural thoracic-abdominal CT scan multiplanar reconstruction (MPR) and 3D volume rendering images. (**A**) TEE image showing the re-entry tear in the aortic intima (**arrow**). (**B**) TEE image with color Doppler of the re-entry tear, (C) TEE image with the delivery system crossing the re-entry tear (**arrowhead**). (**D** and **E**) Simultaneous TEE and fluoroscopic images showing the device disk opened in the false lumen (**arrowhead**). (**F**) Fluoroscopic, (**G**) 3D volume rendering, and CT scan images in (**H**) sagittal, (**I**) transverse, and (**J**) coronal views showing the device fully deployed and the successful re-entry tear closure (**arrowhead**). The volume-rendering image (**G**) reveals the limited protrusion of the GSO occluder disk into the ostium of the left subclavian artery. Ao = aorta; FL = false lumen; TEE = transesophageal echocardiography.

The transapical approach was used in standard fashion,^7^ and the intervention was performed under general anesthesia in the cardiac catheterization laboratory with a team of cardiovascular surgeons and cardiologists. A 12-F, 30-cm-long Check Flow sheath (Cook Medical, Bloomington, IN) was inserted through the apex and the aortic bioprosthesis without noticeable hemodynamic impairment. CT overlay and hybrid navigation using continuous transesophageal echocardiography and fluoroscopy guidance allowed successful identification and crossing of the tear using a 0.89-mm (0.035-inch) Terumo Glidewire Advantage guidewire (Terumo, Tokyo, Japan) through a 5-F Judkins right diagnostic catheter. Subsequently, the 25-mm GSO was carefully and successfully deployed, leading to the immediate and complete exclusion of the proximal part of the false lumen (Fig. 2, A-G).

The fluoroscopy time was 16.32 minutes, and 90 mL of Visipaque was used as a contrast agent for injections in the aortic arch. Angiographic and echocardiographic visualization further validated the accuracy of the device's deployment.

Shortly after the septal occluder’s deployment, feeding into the false lumen stopped with the immediate onset of thrombus formation. Angiographic and echocardiographic monitoring demonstrated good permeability of the true lumen and, thus, the procedure’s success.

A thoracic-abdominal-pelvic CT scan was performed 4 days after the procedure and confirmed the successful closure of the re-entry tear at the distal end of the prosthesis (Fig. 2H). The imaging revealed that the anterior portion of the GSO occluder extended approximately 40% over the origin of the left subclavian artery without clinical relevance. This finding suggests that the procedure effectively secured the prosthesis while maintaining adequate coverage and support at the targeted location. A similar result could be observed 1 month postsurgery on the follow-up CT scan.

We cannot exclude a risk of aorta erosion around the device or recurrent tear. The patient will be followed by an annual CT scan or MRI (no contraindication to MRI with the GSO device). We believe that the risk of device migration is low, with the use of a significantly larger device than the tear. This approach also provides a better distribution of the device pressure on the aorta.

This case highlights the successful transapical closure of a re-entry tear in a residual dissection of the descending thoracic aorta with rapid pseudoaneurysm expansion 3 years after aortic root and hemiarch replacement for acute type A dissection. The use of a custom 3D-printed model for preprocedural planning simulation proved invaluable. Immediate cessation of blood flow into the false lumen and the subsequent thrombosis were observed, thus validating the closure of the re-entry tear. Follow-up imaging demonstrated stable results of the septal occluder’s position with adequate coverage of the dissection tear. This innovative approach may offer a viable option for similar cases, especially when conventional surgical or endovascular treatments are at too high-risk. To our knowledge, this might be the first description of a successful transapical re-entry tear closure with the off-label use of a Gore Cardioform Septal Occluder.

**Novel Teaching Points**

• Transapical closure of a re-entry tear in a residual dissection of the descending aorta with rapid pseudoaneurysm expansion was successful.
• The off-label use of a Gore Cardioform Septal Occluder was safe and efficient under fluoroscopic and TEE guidance.
• A custom 3D-printed model invaluably helped with preprocedural planning and simulation.
